# Efficacy and safety of anemoside B4 in canine pneumonia treatment: a prospective, randomized controlled trial

**DOI:** 10.3389/fvets.2025.1530318

**Published:** 2025-02-12

**Authors:** Jinzhao Ji, Xiaoqing Ding, Chuanli Liu, Lingling Dai, Junting Yu, Linghao Li, Shaobing Wan, Yangyang Song, Junqing Zhao, Zhetong Su, Kun Jia, Shoujun Li

**Affiliations:** ^1^College of Veterinary Medicine, South China Agricultural University, Guangzhou, China; ^2^Guangdong Technological Engineering Research Center for Pet, Guangzhou, China; ^3^Guangxi Innovate Pharmaceutical Co., Ltd, Guangxi, China

**Keywords:** anemoside B4, canine pneumonia, randomized controlled trial, inflammation, phytomedicine

## Abstract

**Background:**

Canine pneumonia is a serious respiratory disease often associated with Canine Infectious Respiratory Disease (CIRD). Current treatment strategies primarily rely on antibiotics and corticosteroids; however, the emergence of antibiotic resistance and potential side effects from prolonged corticosteroid use limit the effectiveness of these therapies in clinical practice. These challenges highlight the urgent need for alternative treatments. Anemoside B4 (AB4), derived from the traditional Chinese medicine *Pulsatilla*, has shown promise in preclinical studies for modulating inflammatory responses and improving clinical symptoms of pneumonia. Therefore, AB4 may offer a valuable alternative treatment option for canine pneumonia in veterinary medicine.

**Methods:**

A prospective, randomized controlled trial was conducted at the Veterinary Drug Research and Evaluation Center of South China Agricultural University. Seventy-two dogs with mild-to-moderate pneumonia were randomly assigned to one of three groups: AB4, placebo, or Chuanxinlian injection (CXL). The primary outcome was the effect of AB4 on comprehensive clinical scoring of canine pneumonia; secondary outcomes included recovery times for primary symptoms and efficacy assessments. Additionally, AB4′s safety in clinical applications was evaluated.

**Results:**

The AB4 group demonstrated significantly lower composite clinical scores on Days 7 and 14 compared to the placebo group (*p* = 0.033 and *p* = 0.000, respectively). Significant differences in recovery times for fever and dyspnea were observed between the AB4 and placebo groups (*p* = 0.041 and *p* = 0.024, respectively). Moreover, the cure rate and overall efficacy on Day 14 were significantly higher in the AB4 group than those in the placebo group (*p* = 0.001 and *p* = 0.009, respectively).

**Conclusion:**

These findings suggest that AB4 may be a promising treatment option for canine pneumonia, potentially serving as an alternative to traditional therapies. Further research is needed to explore its clinical potential in veterinary medicine.

## 1 Introduction

Canine pneumonia is a serious respiratory disease commonly observed in dogs with Canine Infectious Respiratory Disease (CIRD). This condition typically begins with a viral infection of the upper respiratory tract, with common pathogens including canine adenovirus type 2 (CAV-2), canine influenza virus (CIV), and canine herpesvirus type 1 [CHV-1; (1)]. These pathogens spread rapidly in crowded or stressful environments, such as animal shelters and breeding facilities (2). Viral infections can lead to bronchopneumonia or bronchointerstitial pneumonia. As the disease progresses, damage to the respiratory epithelium and excessive inflammation further compromise the lungs' natural defenses, increasing the risk of secondary bacterial infections, with common bacterial pathogens including *Streptococcus* and *Bordetella bronchiseptica* (3, 4). Primary clinical signs include coughing, dyspnea, and fever, with symptoms potentially lasting for several days to weeks. Current treatment strategies primarily rely on antibiotics and corticosteroids; however, concerns over antibiotic resistance and potential side effects from prolonged corticosteroid use necessitate the exploration of new therapeutic strategies (5–8).

Anemoside B4 (AB4), an active compound extracted from the traditional Chinese medicine *Pulsatilla*, has demonstrated significant anti-inflammatory and immunomodulatory effects (9–13). AB4 modulates the NF-κB and TLR4/MyD88 signaling pathways, inhibiting the release of pro-inflammatory cytokines such as TNF-α, IL-6, and IL-1β (14, 15). Additionally, AB4 reduces the expression of IL-12 and STAT4, thereby decreasing Th1 cell activation while simultaneously enhancing the expression of IL-4 and STAT6 to promote Th2 cell-mediated anti-inflammatory responses (16). Furthermore, AB4 significantly improves the histopathological appearance, lung function, oxidative stress, and protease levels in lung tissue (17). Collectively, these mechanisms mitigate inflammation-induced damage, improve clinical symptoms, and reduce the risk of secondary bacterial infections. In clinical practice, appropriate anti-inflammatory treatments can effectively reduce inflammation, accelerate recovery, and alleviate clinical symptoms in dogs, making them a rational and effective therapeutic strategy.

AB4 exhibits a good safety profile, as demonstrated in a study where mice received continuous intraperitoneal injections of 2.5 g/kg of AB4 for 14 days. No significant effects on body weight or overall appearance were observed, and serum levels of ALT, AST, CREA, and BUN remained unchanged, indicating low toxicity of AB4 (18). Additionally, preliminary unpublished safety trials have shown that AB4 is well-tolerated in canine models.

The primary objective of this study was to assess the effect of AB4 on the comprehensive clinical scoring of canine pneumonia. Secondary objectives included efficacy assessments conducted on Days 7 and 14, as well as the evaluation of recovery times for primary symptoms. We also investigated the safety of AB4 in a clinical setting.

## 2 Materials and methods

### 2.1 Trial design

This prospective, randomized controlled trial evaluated the efficacy of AB4 in treating canine pneumonia. The trial was conducted from March to August 2024 at the Veterinary Drug Research and Evaluation Center of South China Agricultural University (SCAU) and the Veterinary Teaching Hospital of SCAU.

### 2.2 Dogs

#### 2.2.1 Inclusion criteria

The inclusion criteria were dogs aged 1–10 years and weighing 3–20 kg. Clinical manifestations included fever, cough, and dyspnea. Chest radiography revealed patchy or patchy infiltrative shadows or interstitial changes (19, 20). Dogs were required to have a defined epidemiological background, such as a history of residing in high-density environments (e.g., breeding facilities or animal shelters) with cases demonstrating a pattern of clustered outbreaks rather than isolated occurrences. For privately owned dogs must have a history of exposure followed by the onset of symptoms (1). Symptoms were mild and common, and disease conditions were stable.

#### 2.2.2. Exclusion criteria

Dogs with other physiological or pathological conditions or systemic diseases affecting drug pharmacokinetics or evaluation of effect indicators were excluded. {Specific exclusions included: 1. confirmed malignancies tumors or severe comorbidities associated with malignancies; 2. severe renal disease [classified as stage IV according to the International Renal Interest Society (IRIS) Chronic Kidney Disease Guidelines or serum creatinine levels >5.0 mg/dL; (21)]; 3. severe hepatic disease, as determined by the attending veterinarian based on liver enzyme levels and clinical symptoms}, dogs with aggressive or uncooperative behavior, dogs administered other drugs or anti-inflammatory medications with prolonged effects within 2 weeks prior to enrollment, dogs with a history of vomiting or recent anesthesia or diagnosed with aspiration or foreign body pneumonia, and dogs with insufficient or unclear medical history (8).

To ensure animal welfare, any dog could withdraw from the study at any time and receive conventional treatment based on owner request or the veterinarian's judgment. For instance, if there is evidence of bacterial infection or clear signs of such an infection during the study, dog could withdraw from the study and receive empirical antibiotic therapy and symptomatic treatment.

#### 2.2.3 Case collection

To reduce iatrogenic infection risk and minimize confounding variables, a block randomization method was used along with a phased admission strategy. Each phase included recruitment and study intervention periods. Preliminary eligible subjects entered a candidate pool for up to 3 days. Enrollment closed upon completion of each recruitment phase, with candidates selected based on predefined protocol criteria. Following enrollment, subjects began the intervention phase, precluding further enrollment until the conclusion of the trial. After the completion of each phase, subsequent recruitment phases continued until the target sample size was achieved. Continuous hospitalization for all trial participants was required throughout the study for precise administration and daily clinical monitoring. All procedures and forms were reviewed and approved by the appropriate animal clinical research ethics committee (approval number: 2023C058) and complied with ethical guidelines.

### 2.3 Randomization and masking

Dogs meeting inclusion criteria were enrolled and assigned a unique random identification number and group code. The randomization sequence was generated using SAS software version 9.4, with block randomization of three and a 1:1:1 allocation ratio for the AB4, CXL, and placebo groups. A dedicated pharmacist prepared the medications and blinded the syringes. A designated “medication administrator” administered the medication according to the randomized codes. Neither the pharmacist nor the medication administrator was involved in clinical evaluations. The Principal Investigator (PI) and data analysts were blinded to group assignments.

### 2.4 Trial medication

The investigational drug, AB4 for injection (Guangxi Innovate Pharmaceutical Co., Ltd., Guangxi, China), was diluted to 48 mg/mL with normal saline and administered at 20 mg/kg. The control medication, Chuanxinlian injection (Guangxi Innovate Pharmaceutical Co., Ltd., Guangxi, China), was administered at 2 mL per dog. The placebo consisted of a normal saline injection (HFQ Co., Ltd., Jiangsu, China) with a volume equivalent to that of the investigational drug. All injections were administered subcutaneously once daily for seven consecutive days.

### 2.5 Clinical evaluation

The clinical efficacy evaluation system comprised three dimensions: clinical symptoms, laboratory tests, and radiographic scores (22). The clinical symptom score assessed the severity of fever, dyspnea, cough, mental status, sputum production, appetite, and nasal discharge, using a graded scoring system. The laboratory test score assessed the complete blood count (CBC), including white blood cell count (WBC), neutrophil count (NEUT), neutrophil percentage (NEUT%), lymphocyte count (LYM), and lymphocyte percentage (LYM%), with scoring based on reference range criteria. Additionally, the absolute counts of WBC, NEUT, and LYM were logarithmically transformed and compared between groups to observe their changing trends. The radiographic scoring system divided the pulmonary fields on chest X-ray images into six distinct zones, each scored on a six-point scale (23, 24). Additional points were allocated for findings such as pleural effusion, pleural thickening with adhesion, and interstitial lung abnormalities. The final composite clinical score combined the weighted clinical symptom score (50%), laboratory test score (30%), and radiographic score [20%; (22)]. Therapeutic efficacy was evaluated based on score reduction from baseline: clinical cure (reduction ≥ 90%), marked efficacy (70% ≤ reduction < 90%), improvement (30% ≤ reduction < 70%), and ineffective (reduction < 30%). “Symptom recovery time” was defined as the day following the last appearance of specific symptoms. Key symptoms, including fever, dyspnea, and cough, were monitored to determine recovery time.

### 2.6 Study procedures

The study spanned 15 days, with a 7-day treatment period and a 7-day post-treatment observation period. Prior to enrollment, a Case Report Form (CRF) was established to collect dogs' basic information. The day of enrollment was marked as D0 (Day 0), and treatment began on D1 (Day 1), The medication was administered once daily via subcutaneous injection for a consecutive period of 7 days, Observations continued through D14 (Day 14), During the study period, routine clinical examinations were conducted daily on the dogs, which included assessment of mental status, measurement of rectal temperature, and collection of heart rate and respiratory rate data. Clinical Composite Scores were obtained on Assessment Days at D0 (baseline), D4 (mid-treatment), D7 (end of treatment), and D14 (7 days post-treatment). These scores included clinical symptom assessment, radiological examination, and laboratory test. The clinical assessment involved scoring various parameters, including fever, cough, dyspnea, psychological condition, food intake, sputum production, and nasal discharge. Radiological examination was conducted by scoring chest X-ray images. Laboratory test included blood collection and subsequent routine hematological analysis Safety assessments included routine clinical examinations, biochemical blood test conducted only on D0, D7 and D14, and monitoring for Adverse Events (AEs). Dogs had free access to food and water throughout the study.

### 2.7 Statistical analysis

Statistical analyses were conducted using SPSS software (version 25.0; IBM, Armonk, NY, USA). Continuous data with normal distribution were expressed as Mean ± Standard Deviation and compared using one-way analysis of variance (ANOVA). If variance homogeneity was satisfied, Bonferroni *post hoc* comparisons were performed; otherwise, Tamhane's T2 test was applied. Non-normally distributed continuous data were assessed with the Kruskal-Wallis H test, followed by pairwise Mann-Whitney U tests when significant, with α adjusted for multiple comparisons (e.g., α = 0.05/3 = 0.0167). If no significant difference was found in the Kruskal-Wallis test, results were reported without pairwise comparisons. Categorical data were reported as frequency/percentage (n, %) and analyzed with Pearson's chi-square test or Fisher's Exact Test, depending on sample size. Time-to-event data were assessed using the log-rank test. All tests were two-tailed, with *p* < 0.05 considered statistically significant.

## 3 Results

### 3.1 Baseline characteristics of dogs

**Figure 1:**
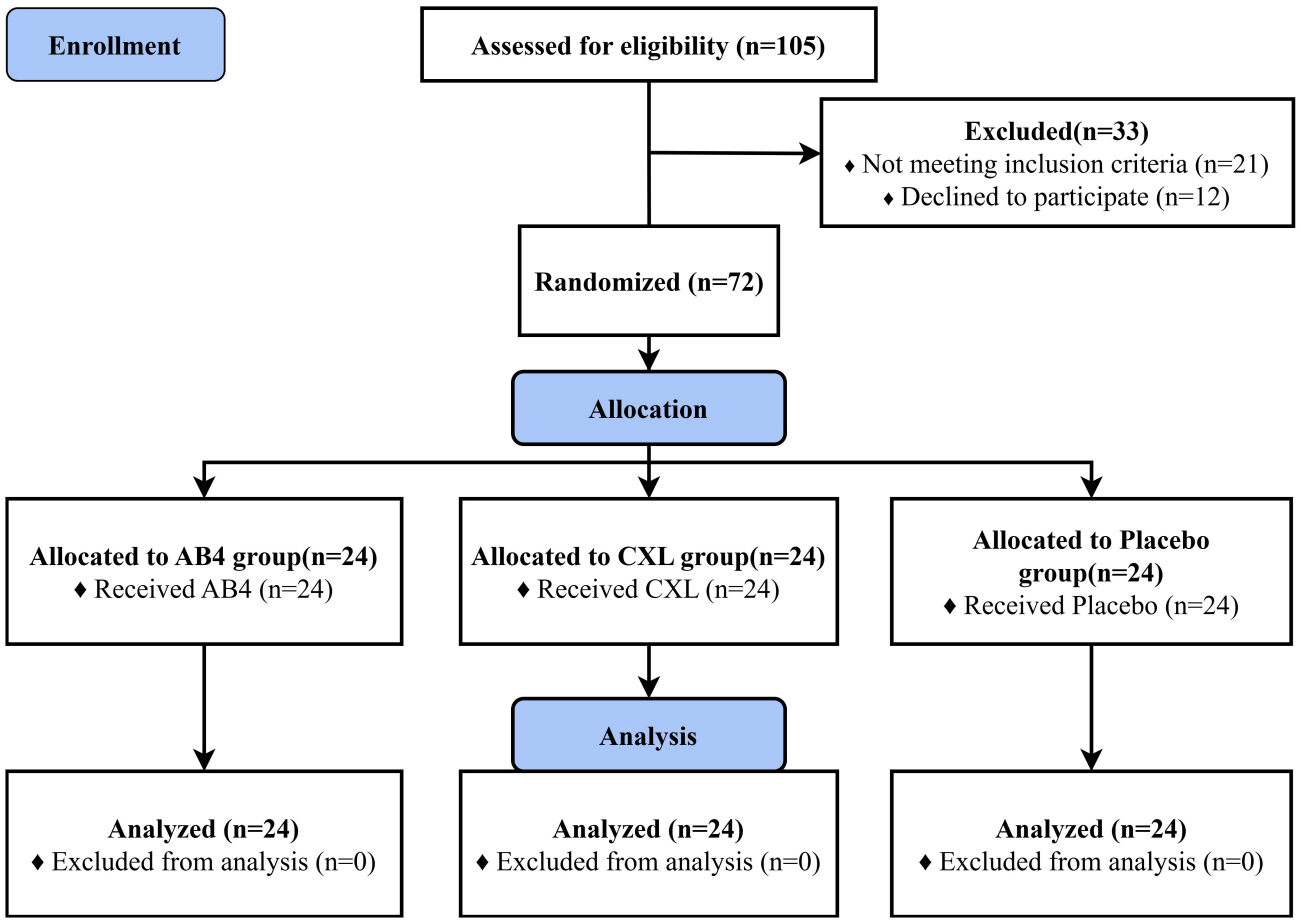
CONSORT flow diagram. AB4, AB4 for injection; CXL, Chuanxinlian injection; Placebo: normal saline injection.

Dogs were recruited through a combination of online promotion and in-person visits. A total of 72 dogs were enrolled (Figure 1, sourced from stray dog shelters (45.83%), dog breeding facilities (25.00%), individual dog traders (15.28%), private owners (11.11%), and dog training centers (2.78%). Statistical analyses and homogeneity testing indicated no statistically significant differences in breed, age, sex, body weight, or pre-treatment composite clinical scores among the AB4, CXL, and placebo groups (*p* > 0.05), confirming the consistency and comparability of baseline characteristics across groups (Table 1). Prior to enrollment, six dogs (five in the AB4 group and one in the CXL group) were diagnosed with ascariasis and received a single oral dose of Drontal^®^ Plus Flavor (KVP Kiel Germany) at a dosage of one tablet per 10 kg of body weight.

**Table 1:** Baseline characteristics.

		**AB4 group (*n* = 24)**	**CXL group (*n* = 24)**	**Placebo group (*n* = 24)**	**Test statistic**	***p*-value**
Breed^a^ [cases (%)]	Chinese rural dog	9 (37.5%)	9 (37.5%)	11 (45.8%)	χ*^2^* = 2.206	0.929
	Mixed breed dog	8 (33.3%)	7 (29.2%)	6 (25.0%)		
	Poodle	3 (12.5%)	2 (8.3%)	1 (4.2%)		
	Other breeds	4 (16.7%)	6 (25.0%)	6 (25.0%)		
Age (y)		2.5 (1, 4)	3 (2, 4.5)	3 (1, 5)	χ*^2^* = 1.825	0.402
Sex [cases (%)]	Male	12 (50.0%)	13 (54.2%)	11 (45.8%)	χ*^2^* = 1.121	0.938
	Female	10 (41.7%)	10 (41.7%)	12 (50.0%)		
	Neutered/spayed	2 (8.3%)	1 (4.2%)	1 (4.2%)		
Body weight (kg)		8.87 ± 3.62	9.67 ± 3.38	9.85 ± 3.20	*F* = 1.121	0.571
Pre-treatment composite clinical scores		10.25 (8, 13)	10.25 (8.7, 13.2)	10.9 (8.8, 12.6)	χ*^2^* = 0.162	0.921

a The top three breeds included in the study were the Chinese Rural Dog, Mixed-Breed Dog, and Poodle, with all the other breeds categorized as Other Breeds.

### 3.2 Clinical symptoms

On Day 0, the dogs presented with clinical signs, including fever, lethargy, tachypnea (abdominal respiration), coughing, expectoration, serous nasal discharge, and reduced or absent appetite. As the disease progressed and treatment was administered, both the AB4 and CXL groups demonstrated effective control of clinical symptom scores, showing gradual alleviation. In contrast, the placebo group exhibited no significant improvement in clinical symptoms during the early phase, with some dogs experiencing further exacerbation. In later stages, a distinct dichotomy in clinical responses emerged: while some dogs exhibited symptom relief, others progressed to severe manifestations characterized by high fever, deep or labored breathing, moderate lethargy, and yellow nasal discharge.

**Figure 2:**
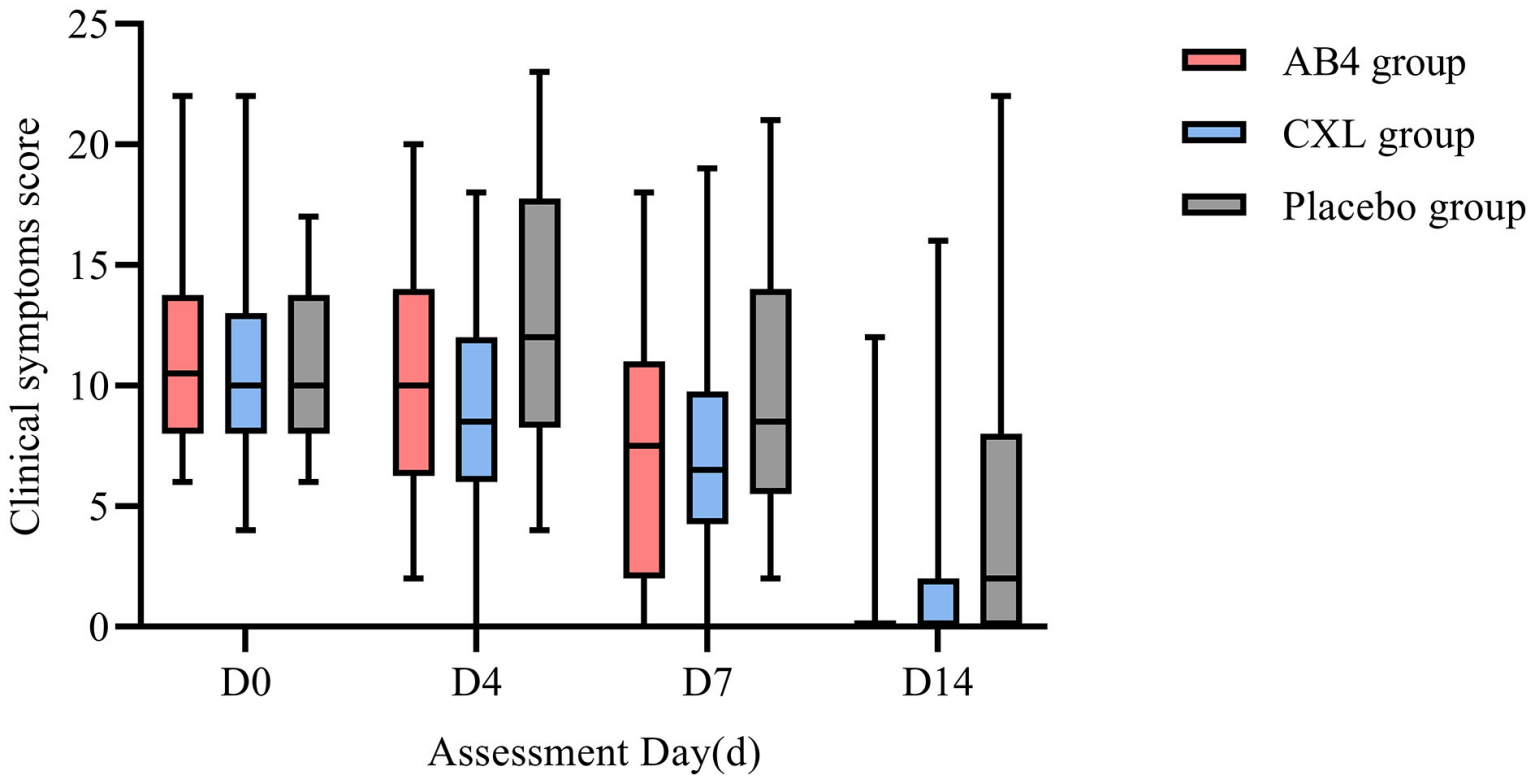
Clinical symptom score on assessment day. AB4, AB4 for injection; CXL, Chuanxinlian injection; Placebo, normal saline injection. Clinical symptom score was evaluated based on the criteria provided in Supplementary Table 1—primary symptoms scoring criteria table and Supplementary Table 2—secondary symptoms scoring criteria table. No asterisk indicates no statistically significant difference between the AB4 and placebo groups (*p* > 0.05).

Statistical analysis of clinical symptom scores on assessment days revealed no significant differences between the AB4, CXL, and placebo groups on D0, D4, D7, and D14 (*p* > 0.05; Figure 2).

### 3.3 Radiographic findings

**Figure 3:**
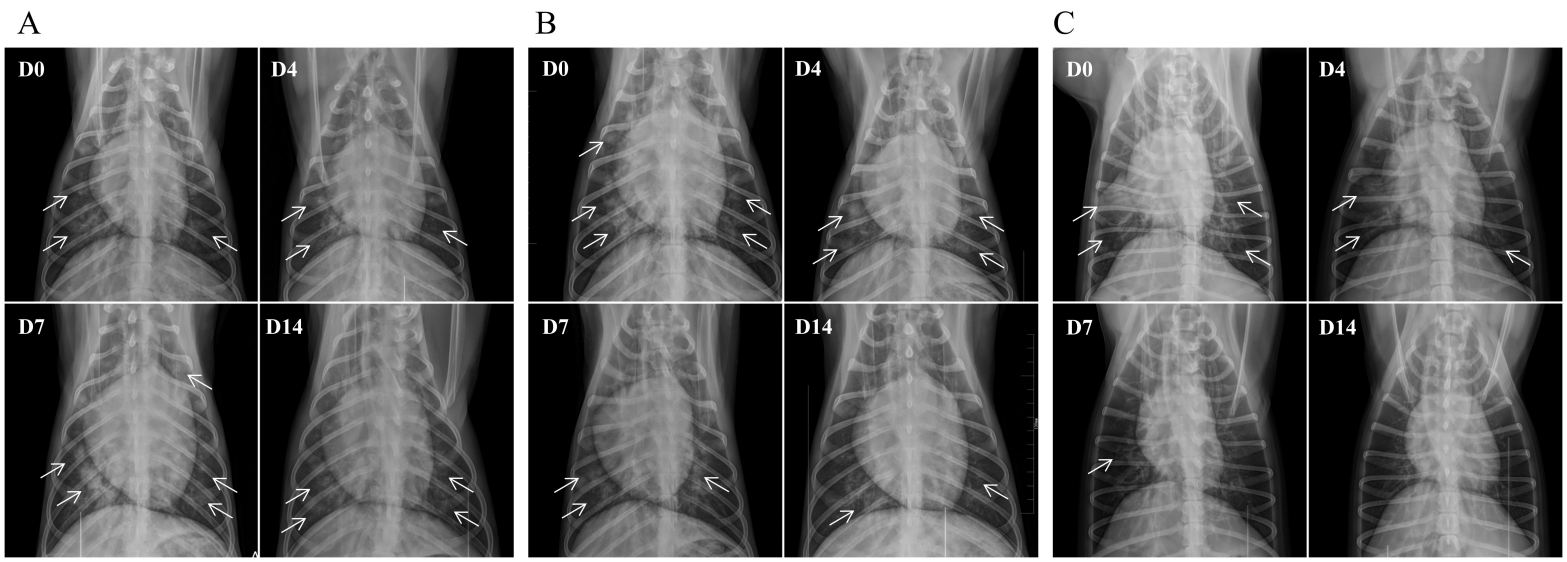
Representative thoracic radiographs of dogs in the AB4, CXL, and placebo groups. **(A)** Placebo group; **(B)** CXL group; **(C)** AB4 group. The radiographs were obtained using a small animal live imaging device (DR) MQD-30R (produced by CFD Srl, Italy) with the following parameters: 70 kVp, 6.3 mA. Dogs were positioned in ventrodorsal recumbency. Arrows indicate areas of pneumonia visible on the radiographs.

On the assessment day, lateral and dorsal recumbent thoracic radiographs were obtained. At enrollment, radiographic findings typically included hilar edema, increased and blurred pulmonary markings, with some dogs presenting patchy infiltrates and air bronchograms. By Day 4 (D4), pulmonary exudation was controlled in the AB4 and CXL groups, with most cases stabilizing, though some displayed worsening radiographic findings. By Day 7 (D7), some dogs in the AB4 and CXL groups entered an absorption phase, with a reduction in areas of increased opacity, while several cases (AB4 group, 7/24; CXL group, 6/24) showed no improvement or worsening. In the placebo group, radiographic changes remained minimal overall, with 14/24 dogs showing further deterioration. By Day 14 (D14), most dogs in the AB4 and CXL groups showed varying degrees of improvement, though residual fibrous streaks or infiltrates persisted in some cases, with a few dogs (AB4 group: 2/24; CXL group: 2/24) showing no significant improvement. In the placebo group, a marked divergence was observed; some dogs entered the absorption phase, whereas others (5/24) showed increased pulmonary exudation, resulting in extensive areas of uniformly increased opacity (Figure 3).

**Figure 4:**
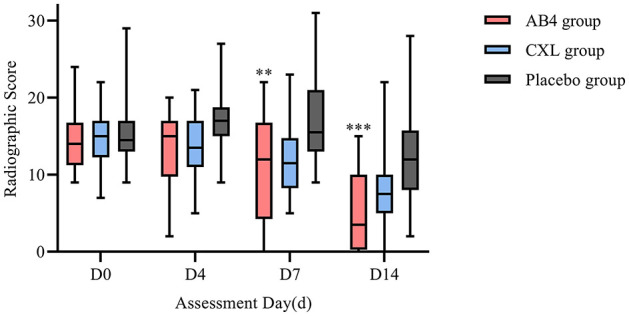
Radiographic scores on assessment day. AB4, AB4 for injection; CXL, Chuanxinlian injection; Placebo, normal saline injection. Radiographic scores were assessed according to the criteria outlined in Supplementary Table 4—lung imaging scoring criteria. **Indicates statistical significance between the AB4 group and the placebo group (*p* < 0.01); ***Indicates *p* < 0.001. No asterisk indicates no statistically significant difference between the AB4 and placebo groups (*p* > 0.05).

Statistical analysis of radiographic scores revealed significantly lower scores in the AB4 group compared to the placebo group on Days 7 and 14, with values of 11.08 ± 7.19 vs. 17.25 ± 5.86 on Day 7 (95% CI: −10.38 to −1.95, *p* = 0.002) and 3.5 (0.5, 10) vs. 12 (8, 15.5) on Day 14 (95% CI: −10.00 to −3.00, *p* = 0.000) in the AB4 and placebo groups, respectively. No significant differences were observed between the AB4 and CXL groups at any time point (*p*>0.05; Figure 4).

### 3.4 Laboratory test

#### 3.4.1 Laboratory test score

**Figure 5:**
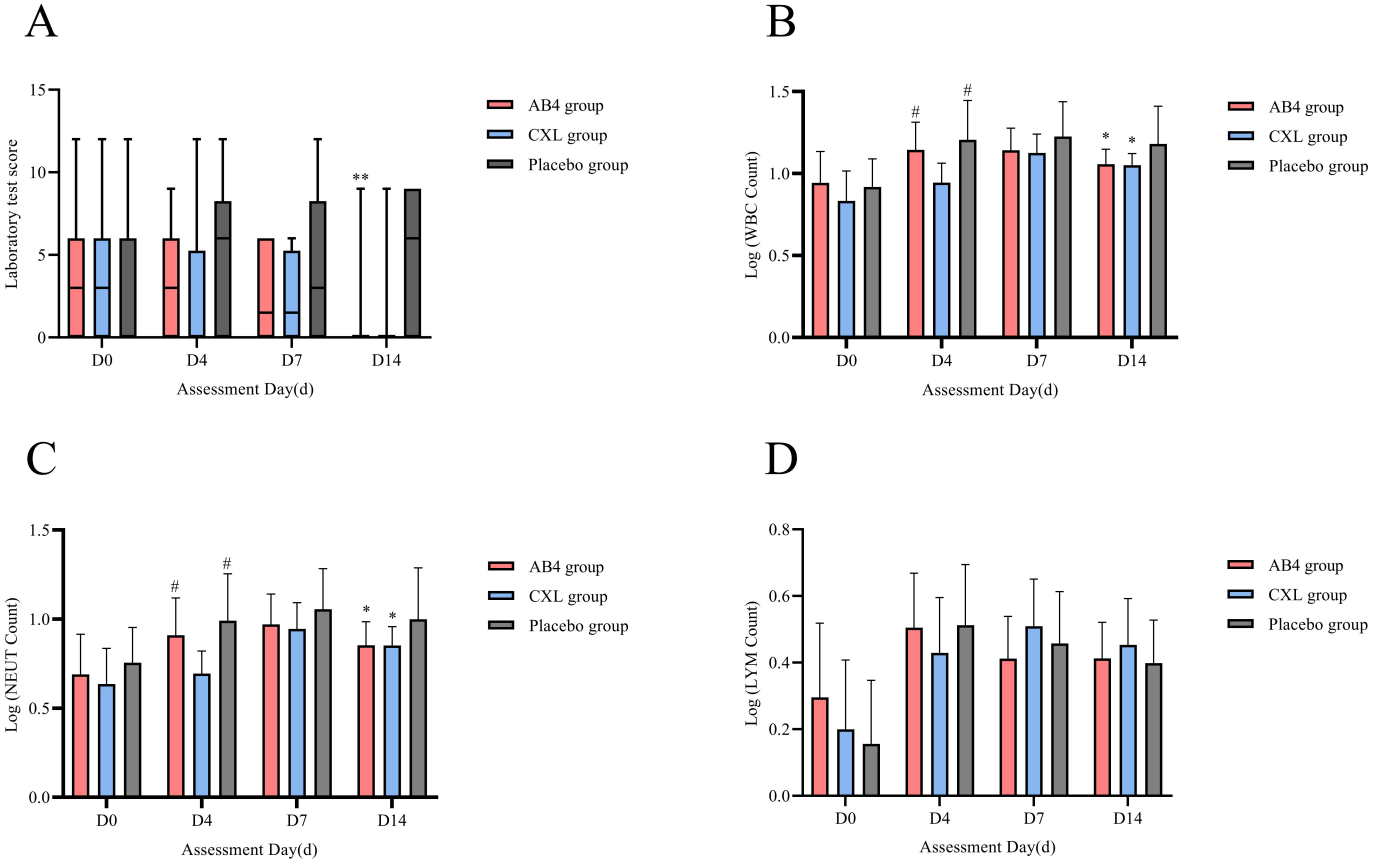
Laboratory test on assessment day. AB4, AB4 for injection; CXL, Chuanxinlian injection; Placebo, normal saline injection. **(A)** Laboratory scores on assessment day. **(B)** Logarithmic comparison of WBC count. **(C)** Logarithmic comparison of NEUT count. **(D)** Logarithmic comparison of LYM count. **(A)** **Indicates statistical significance between the AB4 group and the placebo group (*p* < 0.01); No asterisk indicates no statistically significant difference between the AB4 and placebo groups (*p* > 0.05). **(B–D)** The absolute values of complete blood count (CBC) parameters were log-transformed (logarithm transformation) prior to analysis and compared using ANOVA to ensure suitability for statistical analysis. *Indicates statistical significance compared to Placebo group on the same day (*p* < 0.05), ^#^indicates statistical significance compared to the CXL group on the same day (*p* > 0.05), and data without any markers indicate no statistical significance.

Statistical analysis of laboratory test scores on assessment days (D0, D4, and D7) revealed no significant differences between the AB4 group and the CXL and placebo groups (*p* > 0.05). However, the AB4 group exhibited significantly lower scores compared to the placebo group on Day 14, with values of 0 (0, 0) vs. 6 (0, 9) (95% CI: −6.00 to 0.00, *p* = 0.000), while no significant difference was observed between the AB4 and CXL groups (*p* > 0.05; Figure 5A).

#### 3.4.2 Logarithmic comparison of WBC, NEUT, and LYM

Logarithmic transformation was applied to the absolute counts of WBC, NEUT, and LYM for comparisons. The results showed no statistically significant differences in the logarithmic values of WBC and NEUT between the AB4 group and the Placebo group on Days 0, 4, and 7 (*p* > 0.05). However, on Day 14, the logarithmic value of WBC in the AB4 group was significantly lower than that in the Placebo group (1.06 ± 0.09 vs. 1.18 ± 0.23; 95% CI: −0.23 to −0.02, *p* = 0.016). Similarly, the logarithmic value of NEUT in the AB4 group was significantly lower than that in the Placebo group (0.85 ± 0.13 vs. 1.00 ± 0.29; 95% CI: −0.28 to −0.01, *p* = 0.031). Additionally, no statistically significant differences were observed in the logarithmic values of LYM between the AB4 group and the Placebo group on Days 0, 4, 7, and 14 (*p* > 0.05; Figures 5B–D).

### 3.5 Clinical composite score (weighted)

Statistical analysis of composite clinical scores on follow-up Days 0, 4, 7, and 14 revealed no significant differences between the AB4 and CXL groups (*p* > 0.05). However, on Day 7, a statistically significant difference was observed between the AB4 and placebo groups, with values of 6.61 ± 4.19 vs. 9.71 ± 4.54 (95% CI: −6.01 to −0.19, *p* = 0.033). By Day 14, a significant difference in composite clinical scores persisted between the AB4 and placebo groups, with values of 0.7 (0.1, 2) vs. 4.6 (1.9, 9.1; 95% CI: −5.7 to −1.2, *p* = 0.000).

**Figure 6:**
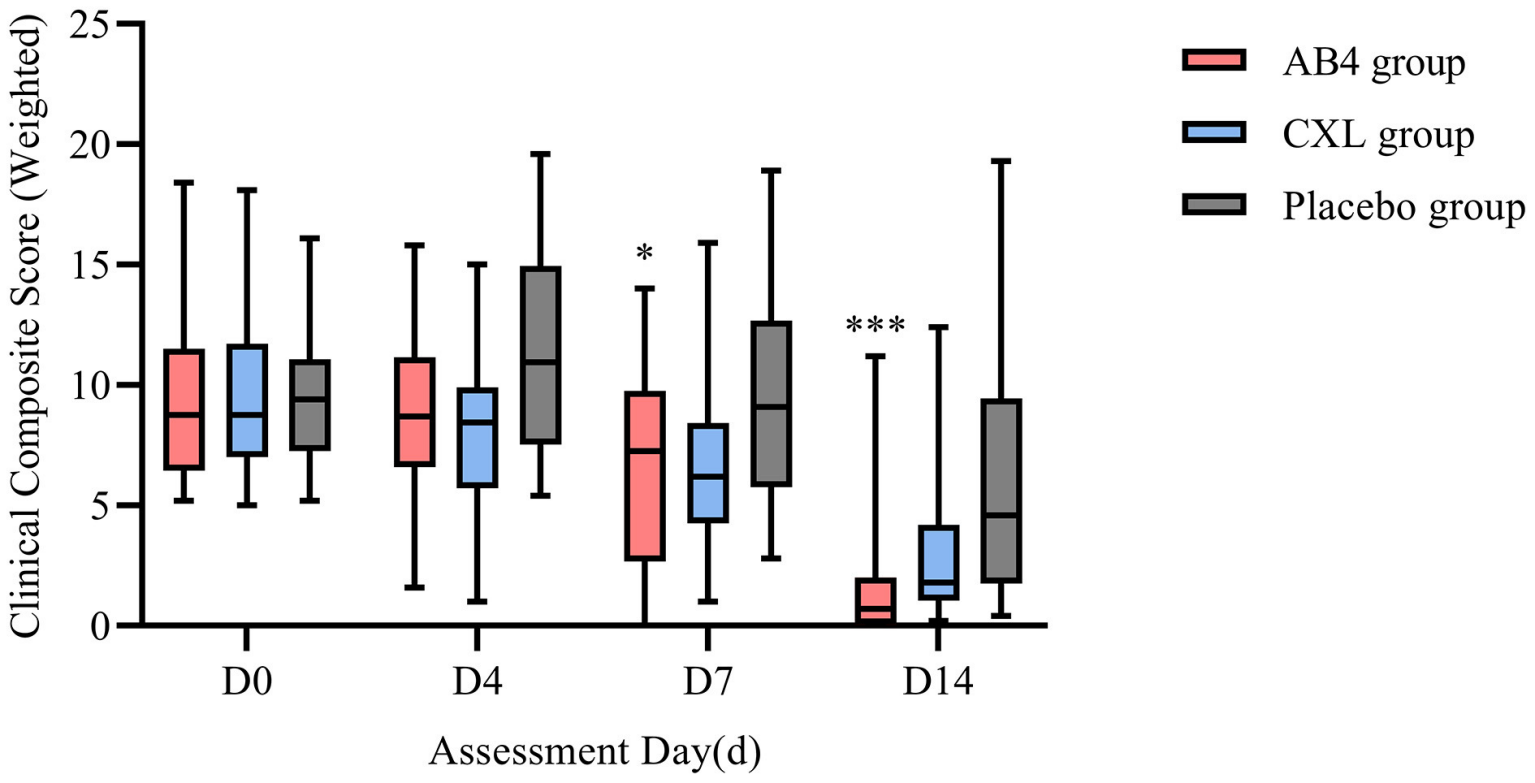
Composite clinical score (weighted) on assessment day. AB4, AB4 for injection; CXL, Chuanxinlian injection; Placebo, normal saline injection. Composite clinical score were weighted according to the criteria outlined in Supplementary Table 5—comprehensive efficacy evaluation weights. *Indicates statistical significance between the AB4 group and the placebo group (*p* < 0.05); ***Indicates *p* < 0.001. No asterisk indicates no statistically significant difference between the AB4 and placebo groups (*p* > 0.05).

Overall, the composite clinical scores for the AB4 and CXL groups revealed a predominantly declining trend over the observation period. However, on Day 14, a minority of cases in both groups exhibited scores exceeding their baseline values (AB4 group: 2/24; CXL group: 2/24). In contrast, the placebo group exhibited an increase in clinical symptom scores on Day 4, and although some cases later showed score reductions, score variability increased over time. On Day 14, seven cases in the placebo group had scores exceeding their baseline values (Figure 6).

### 3.6 Treatment effect indicators

Efficacy evaluations were conducted on Days 7 and 14. On Day 14, the AB4 group demonstrated statistically significant improvements in both cure rate and marked efficacy compared with the placebo group (*p* = 0.001 for cure rate; *p* = 0.003 for marked efficacy). Full details of the efficacy evaluation are presented in Table 2.

**Table 2:** Efficacy evaluations.

**Efficacy evaluation**	**Treatment group**	**Day 7**			**Day 14**		
		**Rate**	***p*** **-value**	χ^2^	**Rate**	***p*** **-value**	χ^2^
Cure rate (%)	AB4	12.50	0.102	Fisher	58.33^*^	—	—
	CXL	0.00			25.00	0.082	3.021
	Placebo	0.00			12.50	0.001	11.021
Marked efficacy (%)	AB4	20.83	0.062	Fisher	70.83^*^	—	—
	CXL	8.33			62.50	0.54	0.375
	Placebo	0.00			33.33	0.009	6.762
Overall response (%)	AB4	54.17	0.248	2.786	87.50	0.083	4.982
	CXL	54.17			83.33		
	Placebo	33.33			62.50		
Non-response (%)	AB4	45.83	0.248	2.786	12.50	0.083	4.982
	CXL	45.83			16.67		
	Placebo	66.67			37.50		

1. Marked Efficacy: Includes cases that are either cured or show significant improvement. Overall Response: Further includes all levels of improvement (cure + marked efficacy + partial improvement).

2. “Fisher” indicates Fisher's Exact Test, used when a chi-square test was not applicable. Similar notation applies to subsequent comparisons.

3. The significance level was adjusted using the Bonferroni correction to α/3 = 0.0167.

4. “^*^” indicates a statistically significant difference compared to the placebo group.

### 3.7 Time to recovery of primary symptoms

**Figure 7:**
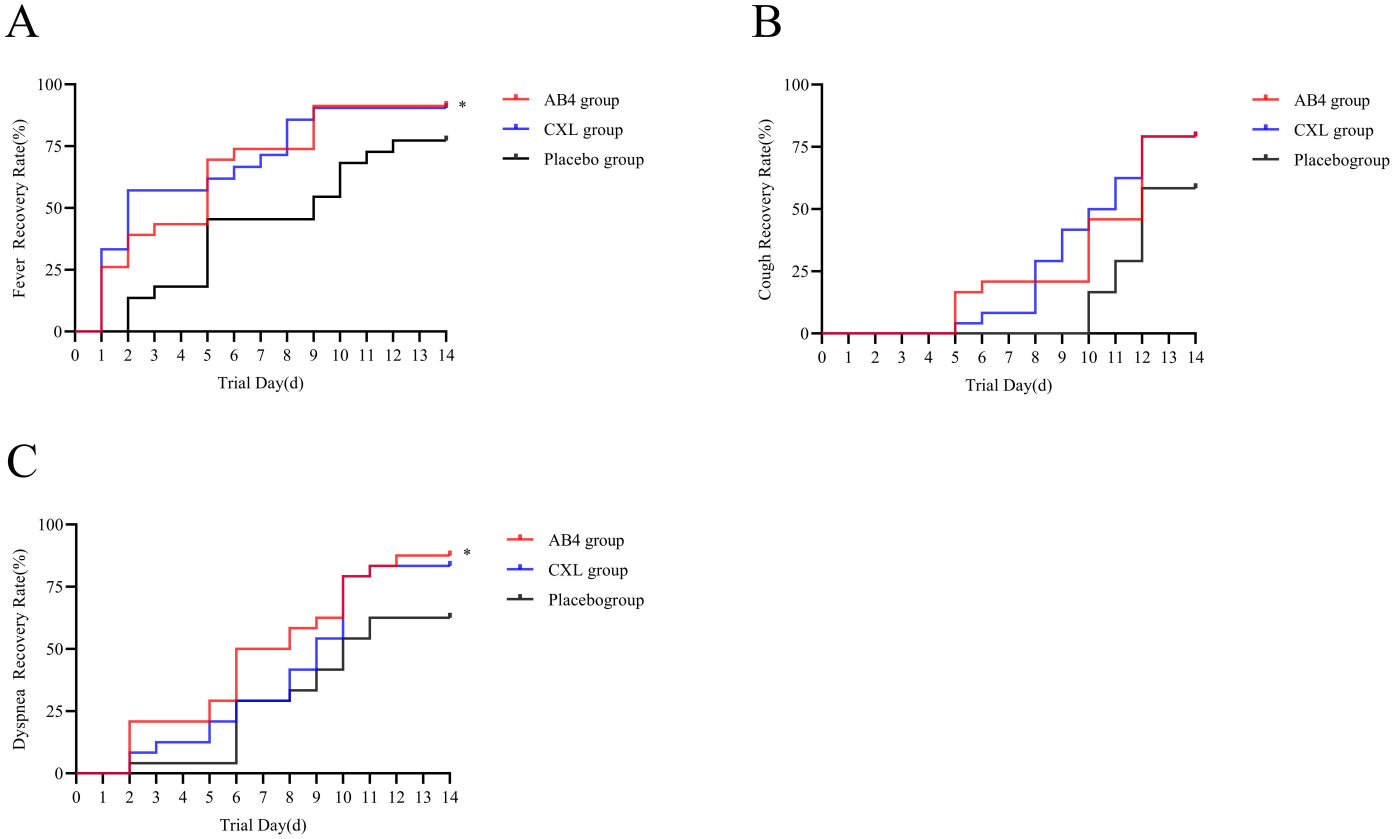
Time to recovery of primary symptoms. **(A)** Time to recovery of fever symptoms. **(B)** Time to recovery of cough symptoms. **(C)** Time to recovery of dyspnea symptoms. The Log-rank test was used to analyze the time to recovery from primary symptoms. Dyspnea (“*”) indicates statistical significance between the AB4 and placebo groups (*p* < 0.05).

Daily monitoring and statistical analysis of recovery times for the primary symptoms of fever, dyspnea, and cough in the AB4, CXL, and placebo groups revealed significant differences in recovery times for fever and dyspnea between the AB4 and placebo groups. Specifically, the median recovery time for fever in the AB4 group was 5 days (95% CI: 3.13 to 6.87 days) compared to 5 days (95% CI: 1.4 to 8.60 days) in the placebo group (*p* = 0.041). For dyspnea, the median recovery time in the AB4 group was 6 days (95% CI: 3.94 to 8.06 days) compared to 10 days (95% CI: 8.09 to 11.91 days) in the placebo group (*p* = 0.024). No significant difference in recovery time for cough was observed between the two groups (*p* = 0.063). Compared with the CXL group, the AB4 group showed no statistically significant differences in recovery times for all symptoms (*p* > 0.05; Figure 7).

### 3.8 Safety

#### 3.8.1 Clinical observations and local tolerance

No instances of near-death or mortality were observed during the trial period. Routine vital signs and physical examinations of the experimental dogs revealed no abnormalities potentially related to the medication. Local tolerance at the injection site was good, with no signs of redness, induration, ulceration, or other abnormal reactions. The experimental dogs did not exhibit behaviors indicative of discomfort, such as scratching, biting, or restlessness.

#### 3.8.2 Adverse events (AEs)

During the study, adverse events, including vomiting, diarrhea, restlessness, and suspected secondary infections, were observed. Statistical analysis revealed no significant differences in the incidence of adverse events among the AB4, CXL, and placebo groups (*p* > 0.05; Table 3).

**Table 3:** Adverse events.

**Adverse event**	**Treatment group**	**Rate (%)**	***p*-value**	**χ^2^**
Vomiting (cases; %)	AB4	1 (4.20%)	1.00	Fisher
	CXL	1 (4.20%)		
	Placebo	0 (0.00%)		
Diarrhea (cases; %)	AB4	2 (8.30%)	1.00	Fisher
	CXL	1 (4.20%)		
	Placebo	2 (8.30%)		
Restlessness (cases; %)	AB4	1 (4.20%)	1.00	Fisher
	CXL	2 (8.30%)		
	Placebo	1 (4.20%)		
Suspected secondary infections (cases; %)	AB4	0 (0.00%)	0.102	Fisher
	CXL	0 (0.00%)		
	Placebo	3 (12.50%)		

#### 3.8.3 Hematological safety analysis

On Day 7, WBC, NEUT, and MON levels in the AB4 group were significantly elevated compared with those at baseline (*p* < 0.025). The placebo group showed significantly higher levels of these markers than the AB4 group on the same day (*p* < 0.05), suggesting a more pronounced inflammatory response in the placebo group, likely due to the absence of treatment. This finding indicated that the increases in WBC, NEUT, and MON observed in the AB4 group were likely typical immune responses to infection or inflammation, rather than drug-induced (25).

On Day 14, RBC, HGB, and HCT levels in the AB4 group showed significant increases from baseline (*p* < 0.025) but no significant differences compared to the placebo group on the same day (*p* > 0.05). Some dogs presented with anemia, malnutrition, and parasitic infections at enrollment. Standardized feeding care and deworming treatments during the study may have contributed to the observed improvements in these parameters (26). Additionally, on Day 14, PLT levels in the AB4 group showed significant changes compared to baseline (*p* < 0.025), with no significant differences compared to the placebo group (*p* > 0.05). Both groups showed an increase in PLT levels from baseline, with a more pronounced increase in the placebo group. This increase is likely related to infection and inflammation, as the body releases large amounts of inflammatory mediators such as IL-6, which stimulate bone marrow platelet production. The stronger inflammatory response in the placebo group, due to the lack of treatment, likely led to increased platelet production (27). Since PLT levels in the AB4 group remained within the reference range, these changes were not considered drug-related. For detailed data, please refer to Supplementary Table 13.

#### 3.8.4 Biochemical safety analysis

On Day 7, ALT, AST, and TBIL levels in the AB4 group showed significant changes compared to those at baseline (*p* < 0.025). On Day 14, TBIL, ALT, UREA, and TP levels in the AB4 group were also significantly different from those at baseline (*p* < 0.025). However, TBIL, ALT, AST, and TP levels remained within reference ranges throughout the study, with no consistent upward or downward trends, suggesting that these changes likely represent normal physiological fluctuations rather than drug-induced, clinically significant liver injury.

Some dogs in the AB4 group had UREA levels below the normal range at enrollment; however, these levels increased by Day 14, with the median and interquartile range falling within reference ranges. The observed changes in UREA may have been due to preexisting nutritional imbalances or insufficient protein intake, leading to lower urea levels (28). Analysis in conjunction with CREA results suggests that AB4 had no significant impact on renal function. For detailed data, please refer to Supplementary Table 14.

## 4 Discussion

In recent years, phytomedicines and their extracts have demonstrated significant efficacy in managing COVID-19 pneumonia, with Lianhua Qingwen capsules and *Andrographis paniculata* extract showing particular effectiveness in reducing inflammation, alleviating symptoms, and shortening treatment duration while also demonstrating good safety profiles (29–31). These findings underscore the potential of traditional Chinese medicine in treating inflammatory diseases. AB4, an emerging plant extract, has been shown in preclinical studies to effectively mitigate inflammatory responses and improve clinical symptoms by modulating inflammatory signaling pathways, thereby reducing the risk of secondary bacterial infections (32–34). Furthermore, AB4′s favorable safety profile and low toxicity in animal models make it a promising option for treating canine pneumonia, particularly considering antibiotic resistance and the side effects associated with corticosteroid use. AB4 holds potential as an effective alternative therapy for dogs with pneumonia, offering promising avenues for future research and clinical practice, thereby promoting its application in veterinary medicine.

AB4 demonstrated antipyretic effects similar to those of its source plant, *Pulsatilla*, and showed greater improvement in dyspnea symptoms than the placebo group (35). Radiographic analyses on Days 7 and 14 revealed significantly lower scores in the AB4 group than in the placebo group, likely due to AB4′s ability to reduce inflammatory cytokine expression, thereby decreasing inflammatory exudation and improving pulmonary pathological changes, ultimately alleviating dyspnea in dogs. However, no significant improvement was observed in cough symptoms. The composite clinical scores of the AB4 group demonstrated a significant decline over the course of the trial, with scores on Days 7 and 14 significantly lower than those of the placebo group, indicating a positive effect of the treatment on the animals' overall condition. No statistically significant difference was observed between the AB4 and CXL groups. The AB4 group also exhibited earlier recovery, with higher cure rates and overall efficacy on Days 7 and 14 compared to the CXL group.

AB4 has been demonstrated to possess significant anti-inflammatory effects. Previous studies have shown that AB4 alleviates pneumonia induced by Klebsiella pneumoniae and influenza virus FM1 in mice by inhibiting the TLR4/MyD88 signaling pathway (15). Additionally, it prevents acute lung injury (ALI) by blocking NLRP3 inflammasome activation (14). These studies consistently report that AB4 reduces the expression of pro-inflammatory cytokines such as TNF-α, IL-6, and IL-1β in blood and lung tissues, thereby decreasing WBC and NEUT counts. In our study, the log-transformed values of WBC and NEUT in the AB4 group were significantly lower than those in the placebo group only on D14, with no significant differences observed at earlier time points. This discrepancy may be attributed to the greater exposure to confounding factors in clinical cases compared to laboratory conditions, such as dogs' physical condition and differences in infectious pathogens. Additionally, the relatively small sample size may have limited the ability to detect statistical differences. However, comparisons of the median absolute counts and mean log-transformed values on D4 and D7 consistently showed lower levels in the AB4 group compared to the placebo group. Although these differences did not reach statistical significance at these time points, the observed downward trends in WBC and NEUT align with previous findings, suggesting that AB4 may exert a potential regulatory effect on the inflammatory response in canine pneumonia.

This study had some limitations. First, the investigation relied on a single drug, with a single-factor design implemented to minimize confounding variables and accurately assess the drug's therapeutic effects. Given that this plant-based drug is in the early stages of research, such an approach is essential for evaluating its efficacy. Future studies should incorporate conventional symptomatic therapies to comprehensively explore the overall therapeutic benefits. Moreover, most cases were sourced from breeding facilities and animal shelters. Future research should include a broader range of subjects, particularly client-owned dogs, to enhance the generalizability of the findings. By expanding the study population, Conducting epidemiological surveys and measuring the concentrations of inflammatory factors in blood samples, we can gain a comprehensive insight into the efficacy of AB4 across various clinical settings.

## 5 Conclusion

In the clinical treatment of canine pneumonia, AB4 for injection effectively alleviated symptoms of fever and dyspnea, shortened the recovery period, improved pulmonary radiographic findings, and reduced comprehensive efficacy evaluation scores, reflecting its therapeutic potential. The observed cure rates on Day 7 and Day 14 were 12.5 and 58.33%, respectively. No serious adverse reactions or mortality were observed in dogs treated with injectable AB4, demonstrated a favorable safety profile.

Future studies should stratify cases based on pneumonia etiology and incorporate conventional treatments alongside AB4 to assess their combined effects. Additionally, together with expanding the study population and inflammatory biomarker analysis, will provide comprehensive insights into AB4′s role as a complementary treatment in diverse veterinary clinical scenarios, potentially enhancing overall clinical outcomes.

## Data Availability

The original contributions presented in the study are included in the article/Supplementary material, further inquiries can be directed to the corresponding author.
