# Supplementary material for: Efficacy and safety of anemoside B4 in canine pneumonia treatment: a prospective, randomized controlled trial
Source: Front Vet Sci. 2025 Feb 12;12:1530318. doi: 10.3389/fvets.2025.1530318 (PMC11861352; doi:10.3389/fvets.2025.1530318)
Supplement: Supplementary file 1 [file Data_Sheet_1.pdf]

# *Supplementary Material*

## 1 Supplementary Table 1- Primary Symptoms Scoring Criteria Table

| Items | Primary Symptoms Scoring Criteria | | | |
|---|---|---|---|---|
| | 0 point | 2 points | 4 points | 6 points |
| Fever | Normothermia | 39℃＜ temperature≤40℃ | 40℃＜ temperature≤41℃ | 41℃＜ temperature≤42℃ |
| Dyspnea | None | Presence of mild shortness of breath (brisk breathing)/mild chest and abdominal breathing (depth of breath slightly above normal) | Visible abdominal/thoracic -abdominal breathing; visible deepening/exertion of breathing | Respiratory failure with mechanical ventilation |
| Cough | None | Lower frequency (occasional) | Frequency general ( 5-15 times/min) | Higher frequency( 1-5 times/min) |

## 2 Supplementary Table 2- Secondary Symptoms Scoring Criteria Table

| Items | Secondary Symptoms Scoring Criteria | | | |
|---|---|---|---|---|
| | 0 point | 1 points | 2 points | 3 points |
| Psychological condition | Normal spirit, both eyes are flexible, bright, agile, wagging tail from time to time | Mildly depressed, with hazel eyes, slow movements, and a tendency to lie down and be lazy | Moderate depression, closed eyes, diminished perception, slowed movement | Severe depression with impaired consciousness, slowness of perception, coma and drowsiness |
| Food intake | Normal feed intake | Reduction in feed intake by less than | Reduction in feed intake by more | Cessation of feeding |

| | | | | |
|---|---|---|---|---|
| | | half. | than half. | |
| Sputum coughing | None | — | Present | — |
| Nasal discharge | None | — | Present | — |

**3 Supplementary Table 3- Laboratory Test Indicator Scoring Scale**

| Items | Grading Standard | |
|---|---|---|
| | 0 points | 3 points |
| WBC | Normal | Abnormal |
| NEUT# | Normal | Abnormal |
| NEUT% | Normal | Abnormal |
| LYM# | Normal | Abnormal |
| LYM% | Normal | Abnormal |

**4 Supplementary Table 4- Lung Imaging Scoring Criteria**

| Parts/ Items | | Grading Standard |
|---|---|---|
| lungs | Left side of upper lung field | Grade 0: no signs of pneumonia (0 points) |
| | Right side of upper lung field | Grade I: slightly heavier or increased lung texture (1 point) |
| | Left side of lung | Grade II (mild): thickening and blurring of the lung texture, or |

| | | |
|---|---|---|
| | center field | fibrous streaks (2 points) |
| | Right side of lung center field | Grade III (moderate): patchy infiltrative shadows, slightly increased density (3 points) |
| | Left side of lower lung field | Grade IV (severe): patchy infiltrative shadows with increased density (4 points) |
| | Right side of the lower lung field | Grade V (very severe): large patchy homogeneous hyperdense shadow (5 points) |
| Pleural effusion | | None (0 points); small amount (blunted rib diaphragm angle) (1 point); large amount (2 points) |
| Pleural thickening adhesions | | None (0 points); Present (2 points) |
| mesenchymal change | | None (0 points); Present (ground glass or lattice shadow) (2 points) |
| Totals | | |

1. Dogs were subjected to chest radiographs in the ventrodorsal recumbency and lateral positions, and the lung radiographs were evaluated in the form of a zonal score, which was divided into the left upper lung field, the right upper lung field, the left middle lung field, the right middle lung field, the left lower lung field, and the right lower lung field. The area of each field of view was scored on a 0 to V scale. The degree of pleural effusion, pleural thickening and adhesion, and interstitial changes were also graded and evaluated, and the sum of the scores of all items was used as the totals.

## 5 Supplementary Table 5- Comprehensive Efficacy Evaluation Weights

| Serial Number | Items | Weights (%) |
|---|---|---|
| 1 | Clinical Symptom Score | 50 |
| 2 | Laboratory Test Score | 30 |
| 3 | Radiographic Score | 20 |

1. Composite Efficacy Score = Clinical Symptom Score * 50% + Laboratory Test Score * 30% + Radiographic Score * 20%

## 6 Supplementary Table 6- Clinical Symptom Score Results on assessment days

| Assessment day | D0 | D4 | D7 | D14 |
|---|---|---|---|---|
| AB4 group | 10.5(8,13.5) | 8.5(6,12) | 7.5(2,11) | 0(0,0) |
| CXL group | 10(8,13) | 10(6.5,14) | 6.5(4.5,9.5) | 0(0,2) |
| Placebo group | 10(8,13.5) | 12(8.5,17.5) | 8.5(6,14) | 2(0,8) |
| Test Statistics | $\chi^2$=0.147 | $\chi^2$=4.676 | $\chi^2$=2.129 | H=5.919 |
| *p* value | 0.864 | 0.012 | 0.127 | 0.052 |

## 7 Supplementary Table 6.1- Clinical Symptom Score Results with post-hoc comparisons of Day 4

| Group | 95% CI lower bound | 95% CI upper bound | *p* value |
|---|---|---|---|
| AB4 group-CXL group | -2.071 | 4.571 | 1.000 |
| CXL group-Placebo group | 0.721 | 7.362 | 0.012 |
| AB4 group-Placebo group | -6.112 | 0.529 | 0.129 |

1.post-hoc comparisons were performed using the Bonferroni method.

## 8 Supplementary Table 7- Radiographic Score Results on assessment days

| Assessment day | D0 | D4 | D7 | D14 |
|---|---|---|---|---|
| AB4 group | 14(11.5,16.5) | 15(10.5,17) | 12(4.5,16.5)** | 7.5(5,10)*** |

| | | | | |
|---|---|---|---|---|
| CXL group | 15(12.5,17) | 13.5(11,17) | 11.5(8.5,14.5) | 3.5(0.5,10) |
| Placebo group | 14.5(13,17) | 17(15,18.5) | 15.5(13,21) | 12(8,15.5) |
| Test Statistics | H=1.078 | H=6.694 | *F*=7.585 | H=15.202 |
| *p* value | 0.583 | 0.035 | 0.001 | 0 |

1. The mark "***" indicates that the AB4 group is statistically significant from the Placebo group ($p < 0.001$).

## 9 Supplementary Table 7.1- Radiographic Score Results with post-hoc comparisons of Day 4

| Group | 95% CI lower bound | 95% CI upper bound | *p* value |
|---|---|---|---|
| AB4 group-CXL group | -3.00 | 3.00 | 0.788 |
| CXL group-Placebo group | -6.00 | -1.00 | 0.015 |
| AB4 group-Placebo group | -6.00 | 0.00 | 0.045 |

1.post-hoc comparisons were performed using the Mann-Whitney U test, with α adjusted for the number of comparisons (α=0.05/3=0.0167).

## 10 Supplementary Table 7.2- Radiographic Score Results with post-hoc comparisons of Day 7

| Group | 95% CI lower bound | 95% CI upper bound | *p* value |
|---|---|---|---|
| AB4 group-CXL group | -3.383 | 5.049 | 1.000 |
| CXL group-Placebo group | -9.549 | -1.118 | 0.008 |
| AB4 group-Placebo group | -10.383 | -1.951 | $0.002^{**}$ |

1.post-hoc comparisons were performed using the Bonferroni method.

2. The mark “**” indicates that the AB4 group is statistically significant from the Placebo group ($p < 0.01$).

## 11 Supplementary Table 7.3- Radiographic Score Results with post-hoc comparisons of Day 14

| Group | 95% CI lower bound | 95% CI upper bound | *p* value |
|---|---|---|---|
| AB4 group-CXL group | -1.00 | 5.00 | 0.13 |
| CXL group-Placebo group | -7.00 | -2.00 | 0.004 |
| AB4 group-Placebo group | -10.00 | -3.00 | 0.000*** |

1.post-hoc comparisons were performed using the Mann-Whitney U test, with α adjusted for the number of comparisons (e.g., α=0.05/3=0.0167).

2. The mark “***” indicates that the AB4 group is statistically significant from the Placebo group ($p < 0.001$).

## 12 Supplementary Table 8- Laboratory Test Score Results on assessment days

| Assessment day | D0 | D4 | D7 | D14 |
|---|---|---|---|---|
| AB4 group | 3(0,6) | 3(0,6) | 1.5(0,6) | 0(0,0)*** |
| CXL group | 3(0,6) | 0(0,4.5) | 1.5(0,4.5) | 0(0,0) |
| Placebo group | 0(0,6) | 6(0,7.5) | 3(0,7.5) | 6(0,9) |
| Test Statistics | H=0.459 | H=0.5.166 | H=3.167 | H=17.489 |
| *p* value | 0.795 | 0.076 | 0.205 | 0 |

1.post-hoc comparisons were performed using the Bonferroni method.

2.The mark "***" indicates that the AB4 group is statistically significant from the Placebo group (p < 0.001).

## 13 Supplementary Table 8.1- Laboratory Score Results with post-hoc comparisons of Day 4

| Group | 95% CI lower bound | 95% CI upper bound | *p* value |
|---|---|---|---|
| AB4 group-CXL group | 0.00 | 0.00 | 0.712 |
| CXL group-Placebo group | -6.00 | 0.00 | 0.001 |
| AB4 group-Placebo group | -6.00 | 0.00 | 0.000*** |

1.post-hoc comparisons were performed using the Mann-Whitney U test, with α adjusted for the number of comparisons (α=0.05/3=0.0167).

2.The mark "***" indicates that the AB4 group is statistically significant from the Placebo group (*p* < 0.001).

## 14 Supplementary Table 8.2- The absolute counts and log-transformed values of WBC, NEUT, and LYM

| Assessment day | Group | WBC ($\times 10^9 \cdot L^{-1}$) | NEUT ($\times 10^9 \cdot L^{-1}$) | LYM ($\times 10^9 \cdot L^{-1}$) | Log-WBC | Log-NEUT | Log-LYM |
|---|---|---|---|---|---|---|---|
| Day 0 | AB4 | 8.39(6.5,10.64) | 4.51(3.21,6.42) | 2.01(1.66,2.53) | 0.94±0.19 | 0.69±0.23 | 0.3±0.22 |
| | CXL | 6.91(4.77,9.02) | 4.49(3.03,6.36) | 1.54(1.28,1.95) | 0.83±0.18 | 0.64±0.2 | 0.2±0.21 |
| | Placebo | 8.2(5.96,11.48) | 5.64(3.78,7.73) | 1.62(1.07,1.92) | 0.92±0.17 | 0.76±0.2 | 0.16±0.19 |
| Day 4 | AB4 | 12.81(10.34,17.48) | 7.23(5.37,10.59) | 3.64(2.91,4) | 1.14±$0.17^{\#}$ | 0.91±$0.21^{\#}$ | 0.5±0.16 |

| | | | | | | | |
|---|---|---|---|---|---|---|---|
| | CXL | 8.69(7.33,11.16) | 4.96(4.31,6.17) | 2.78(1.91,3.26) | 0.94±0.12 | 0.69±0.13 | 0.43±0.17 |
| | Placebo | 16.16(9.67,28.46) | 8.32(5.89,18.99) | 3.43(2.65,4.3) | 1.21±0.24# | 0.99±0.26# | 0.51±0.18 |
| | AB4 | 14.62(10.1,18.51) | 10.02(6.61,14) | 2.81(2.19,3.34) | 1.14±0.14 | 0.97±0.17 | 0.41±0.13 |
| Day 7 | CXL | 13.87(10.51,15.91) | 9.11(6.68,10.93) | 3.2(2.54,4.31) | 1.13±0.12 | 0.94±0.15 | 0.51±0.14 |
| | Placebo | 17.63(10.28,23.82) | 10.36(7.18,16.81) | 3(2.31,3.65) | 1.23±0.21 | 1.06±0.23 | 0.46±0.16 |
| | AB4 | 11.06(9.75,14.26) | 7.27(5.48,9.66) | 2.45(2.26,2.95) | 1.06±0.09* | 0.85±0.13* | 0.41±0.11 |
| Day 14 | CXL | 11.02(9.92,12.78) | 7.38(6.4,8.27) | 3.03(2.22,3.39) | 1.05±0.07* | 0.85±0.11* | 0.45±0.14 |
| | Placebo | 14.8(10.22,22.46) | 10.66(6.08,15.77) | 2.49(2.26,2.71) | 1.18±0.23 | 1±0.29 | 0.4±0.13 |

1. The absolute values of complete blood count (CBC) parameters were log-transformed (logarithm transformation) prior to analysis and compared using ANOVA to ensure suitability for statistical analysis.

2. An asterisk ("*") indicates statistical significance compared to Placebo group on the same day ($p < 0.05$), a hashtag ("#") indicates statistical significance compared to the CXL group on the same day ($p > 0.05$), and data without any markers indicate no statistical significance.

## 15 Supplementary Table 9- Composite Efficacy Score(Weighted) Results on assessment days

| Assessment day | D0 | D4 | D7 | D14 |
|---|---|---|---|---|
| AB4 group | 8.75(6.5,11.5) | 8.7(6.8,10.9) | 7.25(2.85,9.6) | 0.7(0.1,2) |
| CXL group | 8.75(7,11.65) | 8.45(5.75,9.8) | 6.2(4.3,8.35) | 1.8(1.1,4) |

| | | | | |
|---|---|---|---|---|
| Placebo group | 9.4(7.3,10.85) | 10.95(7.85,14.7) | 9.1(6,12.55) | 4.6(1.9,9.1) |
| Test Statistics | H=0.09 | *F*=5.599 | *F*=4.238 | H=15.599 |
| *p* value | 0.956 | 0.006 | 0.018 | 0.000 |

**16 Supplementary Table 9.1- Composite Efficacy Score(Weighted) Results with post-hoc comparisons of Day 4**

| Group | 95% CI lower bound | 95% CI upper bound | *p* value |
|---|---|---|---|
| AB4 group-CXL group | -3.444 | 1.702 | 1.000 |
| CXL group-Placebo group | -5.952 | -0.806 | 0.006 |
| AB4 group-Placebo group | -5.081 | 0.065 | 0.058 |

1.post-hoc comparisons were performed using the Bonferroni method.

**17 Supplementary Table 9.2- Composite Efficacy Score(Weighted) Results with post-hoc comparisons of Day 7**

| Group | 95% CI lower bound | 95% CI upper bound | *p* value |
|---|---|---|---|
| AB4 group-CXL group | -2.665 | 3.148 | 1.000 |
| CXL group-Placebo group | -5.765 | 0.048 | 0.055 |
| AB4 group-Placebo group | -6.006 | -0.194 | 0.033* |

1.post-hoc comparisons were performed using the Bonferroni method.

2.Marking "*" indicates statistical significance of AB4 group versus Placebo group ($p < 0.05$).

## 18 Supplementary Table 9.3- Composite Efficacy Score(Weighted) Results with post-hoc comparisons of Day 14

| Group | 95% CI lower bound | 95% CI upper bound | *p* value |
|---|---|---|---|
| AB4 group-CXL group | 0 | 1.6 | 0.037 |
| CXL group-Placebo group | -4.8 | -0.4 | 0.013 |
| AB4 group-Placebo group | -5.7 | -1.2 | 0.000*** |

1. post-hoc comparisons were performed using the Mann-Whitney U test, with α adjusted for the number of comparisons (α=0.05/3=0.0167).

2. The mark "***" indicates that the AB4 group is statistically significant from the Placebo group ($p < 0.001$).

## 19 Supplementary Table 10- Fever Recovery Time analysis results

| Groups | Median | 95% CI lower bound | 95% CI upper bound | chi-square test | Significance |
|---|---|---|---|---|---|
| AB4 group | 5* | 3.13 | 6.87 | — | — |
| CXL group | 2 | 1.07 | 2.93 | 0.12 | 0.729 |
| Placebo group | 5 | 1.40 | 8.60 | 4.161 | 0.041 |

1. Logarithmic Rank Test was used for statistical analysis;

2. Marking "*" indicates that AB4 group and Placebo group are statistically significant, while unmarking indicates no statistical significance.

## 20 Supplementary Table 11- Cough Recovery Time analysis results

| Groups | Median | 95% CI lower bound | 95% CI upper bound | chi-square test | Significance |
|---|---|---|---|---|---|
| AB4 group | 12 | 10.33 | 13.67 | — | — |
| CXL group | 10 | 8.08 | 11.92 | 0.358 | 0.55 |
| Placebo group | 12 | 11.32 | 12.68 | 3.467 | 0.063 |

1. Logarithmic Rank Test was used for statistical analysis;

2. Marking "*" indicates that AB4 group and Placebo group are statistically significant, while unmarking indicates no statistical significance.

## 21 Supplementary Table 12- Dyspnea Recovery Time analysis results

| Groups | Median | 95% CI lower bound | 95% CI upper bound | chi-square test | Significance |
|---|---|---|---|---|---|
| AB4 group | 6* | 3.94 | 8.06 | — | — |
| CXL group | 9 | 7.94 | 10.06 | 0.547 | 0.459 |
| Placebo group | 10 | 8.09 | 11.91 | 5.114 | 0.024 |

1.Logarithmic Rank Test was used for statistical analysis;

2. Marking "*" indicates that AB4 group and Placebo group are statistically significant, while unmarking indicates no statistical significance.

## 22 Supplementary Table 13-Summary Table of Hematology Data

| | Group | Day 0 | Day 7 | Day 14 | Reference range |
|---|---|---|---|---|---|
| WBC（$\times 10^9 \cdot L^{-1}$） | AB4 | 8.39（6.5, 10.64） | 14.62（10.1, 18.51）* | 11.06（9.75, 14.26） | 6.00～17.00 |
| | CXL | 6.91（4.77, 9.02） | 13.87（10.51, 15.91）* | 11.02（9.92, 12.78）* | |

| | | | | | |
|---|---|---|---|---|---|
| | Placebo | 8.2（5.96, 11.48） | 17.63（10.28, 23.82）* | 14.8（10.22, 22.46）* | |
| | AB4 | 4.51（3.21, 6.42） | 10.02（6.61, 14）* | 7.27（5.48, 9.66） | |
| NEUT（×10$^{9}$·L$^{-1}$) | CXL | 4.49（3.03, 6.36） | 9.11（6.68, 10.93）* | 7.38（6.4, 8.27）* | 3.62～12.30 |
| | Placebo | 5.64（3.78, 7.73） | 10.36（7.18, 16.81）* | 10.66（6.08, 15.77）* | |
| | AB4 | 2.01（1.66, 2.53） | 2.81（2.19, 3.34） | 2.45（2.26, 2.95） | |
| LYM（×10$^{9}$·L$^{-1}$) | CXL | 1.54（1.28, 1.95） | 3.2（2.54, 4.31）* | 3.03（2.22, 3.39）* | 0.83～4.91 |
| | Placebo | 1.62（1.07, 1.92） | 3（2.31, 3.65）* | 2.49（2.26, 2.71）* | |
| | AB4 | 0.79（0.45, 0.95） | 1.09（0.78, 1.37）* | 0.9（0.65, 1.07） | |
| MON（×10$^{9}$·L$^{-1}$) | CXL | 0.5（0.38, 0.86） | 0.72（0.5, 0.91） | 0.74（0.57, 0.87） | 0.14～1.97 |
| | Placebo | 0.73（0.51, 0.96） | 1.63（0.66, 2.79）* | 1.28（0.64, 3.06）* | |
| | AB4 | 5.21（4.67, 5.47） | 5.28（4.59, 5.95） | 5.86（5.53, 6.35）* | |
| RBC（×10$^{12}$·L$^{-1}$) | CXL | 5.86（5.08, 6.35） | 5.85（5.41, 6.72） | 6.12（5.51, 6.61） | 5.10～8.50 |
| | Placebo | 5.14（4.8, 6.95） | 5.23（4.88, 6.11） | 5.62（5.26, 6.5） | |

| | | | | | |
|---|---|---|---|---|---|
| | AB4 | 105.5（97.5, 117.25） | 111.5（102, 124.75） | 128（117, 137.25）* | |
| HGB （g·L$^{-1}$） | CXL | 135.5（125.75, 149.5） | 142（136, 157）* | 153.5（138, 163.75） | 110.00～190.00 |
| | Placebo | 114.5（104.5, 164.75） | 118（108, 142） | 130.5（116, 158.5） | |
| | AB4 | 29.25（27.03, 31.7） | 31.3（27.63, 34.35） | 35.95（32.73, 37.9）* | |
| HCT （%） | CXL | 37（33.2, 41.18） | 38.9（37.43, 43.35）* | 41.45（36.68, 44.73） | 33.00～56.00 |
| | Placebo | 31.15（29.3, 42.93） | 33.3（29.58, 38.2） | 36.25（33.7, 42.28） | |
| | AB4 | 134.5（108.5, 258.5） | 143.5（97.25, 202） | 238.5（131, 321.5）$^{*\#}$ | |
| PLT （×10$^9$·L$^{-1}$） | CXL | 242（150.75, 271.5） | 262（202.5, 307.5）* | 245（221, 311.75）* | 117.00～490.00 |
| | Placebo | 259.5（220.25, 290.25） | 269（245.25, 354.5） | 384（306, 561.25）* | |

1.The reference range data were obtained from the Mindray BC-5000VET hematology analyzer.

2.For normally distributed data, a Paired t-test was used, while the Wilcoxon Signed-Rank Test was applied for non-normally distributed data.

3.The Bonferroni correction was applied to adjust the significance level to $p = 0.025$ in order to reduce Type I errors.

4.An asterisk (“*”) indicates statistical significance compared to D0 (paired test), a hashtag (“#”) indicates statistical significance compared to the placebo group on the same day, and data without any markers indicate no statistical significance.

**23 Supplementary Table 14-Summary Table of Blood Biochemistry Data**

| | Group | Day 0 | Day 7 | Day 14 | Reference range |
|---|---|---|---|---|---|
| Tbil | AB4 | 0.93(0.75,1.2) | 0.95(0.76,1.25)* | 1(0.71,1.42)*# | 0.00~15.00 |
| | CXL | 1.06(0.59,1.37) | 0.63(0.14,1.1) | 0.57(0.24,0.84) | |
| | Placebo | 0.81(0.5,1.14) | 1.12(0.73,1.52)* | 1.08(0.58,1.34) | |
| ALT | AB4 | 18.7(15.5,31.25) | 17.25(14.4,26.35)* | 17.05(14.1,24.55)*# | 8.00~100.00 |
| | CXL | 49.4(41.05,57.25) | 44.5(37.55,55.25) | 39.65(31.35,43.55)* | |
| | Placebo | 33.1(29.55,40.65) | 33.4(29.8,45.55) | 30.5(26.75,38.05) | |
| AST | AB4 | 14.95(12.9,15.85) | 12.1(9.8,14.2)*# | 14.15(11.65,16.95) | 0.00~50.00 |
| | CXL | 36(28.45,41.6) | 21.25(18.95,35.1) | 21.5(19.6,24.15)* | |
| | Placebo | 19.9(17.8,23.1) | 25(16,36.6)* | 23.5(16.55,28.95) | |
| ALP | AB4 | 93.1(76.4,134.5) | 97.35(87.2,141.7) | 104.9(77.35,132.55) | 23.00~337.00 |
| | CXL | 93.85(67,114.55) | 82.25(56.05,97.85)* | 94.1(60.55,160.05) | |
| | Placebo | 133.7(103.45,198.75) | 154.75(62.35,253.4) | 237.45(146.55,373.1)* | |
| TP | AB4 | 54.21(51.22,57.45) | 57.04(55.08,59.97) | 57.19(56.41,61.4)*# | 48.00~82.00 |
| | CXL | 69.47(65.93,74.43) | 71.72(67.28,75.95) | 67.95(64.54,72.87) | |
| | Placebo | 65.94(54.3,71.45) | 65.63(58.52,72.68) | 67.51(63.41,74.48)* | |
| CRE | AB4 | 48.8(44.1,67.15) | 41(31.45,62.55) | 48.9(41.75,56.25) | 27.0~159.0 |

| | | | | | |
|---|---|---|---|---|---|
| A | CXL | 64.6(58.65,68.7) | 68.25(63.55,72.85) | 60.95(59.15,73.95) | |
| | Placebo | 55.9(30.3,67.15) | 50.25(38.6,65.75) | 45.7(37.25,58.1) | |
| | AB4 | 2.85(2.05,3.27) | 2.79(1.93,4.16) | 3.86(2.93,4.39)$^{*\#}$ | |
| UREA | CXL | 4.3(2.85,6.37) | 4.74(4.2,6.62) | 4.58(4.37,6.5) | 2.50~10.40 |
| | Placebo | 3.92(2.51,5.04) | 3.68(2.32,4.77) | 5.15(3.5,6.34) | |

1.The reference range data were obtained from the Mindray BS-240 automatic biochemical analyzer.

2. For normally distributed data, a Paired t-test was used, while the Wilcoxon Signed-Rank Test was applied for non-normally distributed data.

3. The Bonferroni correction was applied to adjust the significance level to $p = 0.025$ in order to reduce Type I errors.

4. An asterisk (“*”) indicates statistical significance compared to D0 (paired test), a hashtag (“#”) indicates statistical significance compared to the placebo group on the same day, and data without any markers indicate no statistical significance.
